# Effect of Diphenyleneiodonium Chloride on Intracellular Reactive Oxygen Species Metabolism with Emphasis on NADPH Oxidase and Mitochondria in Two Therapeutically Relevant Human Cell Types

**DOI:** 10.3390/pharmaceutics13010010

**Published:** 2020-12-23

**Authors:** Sergejs Zavadskis, Adelheid Weidinger, Dominik Hanetseder, Asmita Banerjee, Cornelia Schneider, Susanne Wolbank, Darja Marolt Presen, Andrey V. Kozlov

**Affiliations:** 1 Ludwig Boltzmann Institute for Experimental and Clinical Traumatology in the AUVA Trauma Research Center, Austrian Cluster for Tissue Regeneration, A-1200 Vienna, Austria; sergejs.zavadskis@trauma.lbg.ac.at (S.Z.); adelheid.weidinger@trauma.lbg.ac.at (A.W.); dominik.hanetseder@trauma.lbg.ac.at (D.H.); asmita.banerjee@trauma.lbg.ac.at (A.B.); cornelia.schneider@trauma.lbg.ac.at (C.S.); susanne.wolbank@trauma.lbg.ac.at (S.W.); darja.marolt@trauma.lbg.ac.at (D.M.P.); 2 Laboratory of Navigational Redox Lipidomics, Department of Human Pathology, IM Seche-Nov Moscow State Medical University, 119146 Moscow, Russia

**Keywords:** diphenyleneiodonium, reactive oxygen species, mitochondria, NADPH-oxidase, differentiation, proliferation

## Abstract

Reactive oxygen species (ROS) have recently been recognized as important signal transducers, particularly regulating proliferation and differentiation of cells. Diphenyleneiodonium (DPI) is known as an inhibitor of the nicotinamide adenine dinucleotide phosphate oxidase (NOX) and is also affecting mitochondrial function. The aim of this study was to investigate the effect of DPI on ROS metabolism and mitochondrial function in human amniotic membrane mesenchymal stromal cells (hAMSCs), human bone marrow mesenchymal stromal cells (hBMSCs), hBMSCs induced into osteoblast-like cells, and osteosarcoma cell line MG-63. Our data suggested a combination of a membrane potential sensitive fluorescent dye, tetramethylrhodamine methyl ester (TMRM), and a ROS-sensitive dye, CM-H2DCFDA, combined with a pretreatment with mitochondria-targeted ROS scavenger MitoTEMPO as a good tool to examine effects of DPI. We observed critical differences in ROS metabolism between hAMSCs, hBMSCs, osteoblast-like cells, and MG-63 cells, which were linked to energy metabolism. In cell types using predominantly glycolysis as the energy source, such as hAMSCs, DPI predominantly interacted with NOX, and it was not toxic for the cells. In hBMSCs, the ROS turnover was influenced by NOX activity rather than by the mitochondria. In cells with aerobic metabolism, such as MG 63, the mitochondria became an additional target for DPI, and these cells were prone to the toxic effects of DPI. In summary, our data suggest that undifferentiated cells rather than differentiated parenchymal cells should be considered as potential targets for DPI.

## 1. Introduction

Diphenyleneiodonium (DPI) is known as a strong inhibitor of the nicotinamide adenine dinucleotide phosphate oxidase (NOX) and also interferes with mitochondrial function. NOX and the mitochondria are two major sources of reactive oxygen species (ROS) in the cell [1,2]. Beside their well-known role as inducers of oxidative stress, ROS have recently also been recognized as important signal transducers, regulating numerous physiological and pathophysiological processes [3]. Consequently, pharmacological treatments targeting ROS have come into focus as possible therapeutic strategies. In this context, DPI has already been tested as a potential drug for several diseases. For example, DPI has been shown to prevent the degeneration of neurons in Parkinson’s disease [4] and show neuroprotective effects after focal cerebral ischemia [5,6]. Another study showed that ultra-low doses of DPI may be a therapeutic agent for the treatment of colitis and colitis-associated colorectal cancer [7]. Furthermore, it has been shown that the activation of NOX may represent a stimulus for cell-cycle activation and that DPI and apocynin inhibit this process in cardiac cells [8]. DPI has also been reported to induce a chemo-quiescence phenotype that potently inhibited the propagation of cancer stem-like cells. This action of DPI was associated with the inhibition of mitochondrial respiration [9]. The interaction of DPI in cancer stem-like cells attracted our attention. Several studies have shown that the inhibition of mitochondrial respiration can increase the production of mitochondrial ROS, which, in turn, can interact with NOX and vice versa (reviewed in [1,10]). Therefore, it can be assumed that DPI could influence different sources of intracellular ROS and orchestrate ROS-dependent processes of cellular metabolism in different cell types.

Human amniotic membrane mesenchymal stromal cells (hAMSCs), as well as bone marrow-derived mesenchymal stromal cells (hBMSCs), are two types of therapeutically relevant stromal cells [11]. However, hAMSCs reside in entirely different niches than hBMSCs and have different functions in vivo. While hBMSCs are multipotent cells [12], hAMSCs are known to express pluripotency markers, such as stage-specific embryonic antigen (SSEA)-3 and SSEA-4 [13]. Of note, considering the ontogenetic development, hAMSCs develop well before the formation of the three germ layers, whereas hBMSCs derive later in embryonic development from the mesodermal lineage [14].

hBMSCs constitute promising therapeutic cells for tissue regeneration, due to their secretory properties and/or their capacity to differentiate [15,16]. It has been shown that the cells change energy metabolism from glycolysis to mitochondrial energy conversion during the process of osteogenic differentiation [17,18].

The aim of this study was to investigate the effects of different DPI concentrations on the ROS metabolism and mitochondrial membrane potential of hAMSCs, undifferentiated hBMSCs, and their osteogenic-induced progeny, as well the osteosarcoma-derived cells (MG63) commonly used for osteoblastic models.

## 2. Materials and Methods

### 2.1. Chemicals and Materials

T75/T175 CELLSTAR^®^ cell culture flasks were purchased from Greiner Bio-One (Kremsmünster, Austria), and 24-well plates were supplied by Corning Inc. (New York, NY, USA). Reagents were obtained from Merck/Sigma-Aldrich (Darmstadt, Germany) unless otherwise noted.

### 2.2. Preparation of Human Amniotic Membrane and Isolation of Human Amniotic Membrane Mesenchymal Stromal Cells

Human placentas were obtained from caesarian sections of term pregnancies with the patient’s full informed consent (Ethikkommission des Landes Oberösterreich, No. 200, 12. 05. 2005) of approval and processed as described previously [19,20]. In brief, placentas were washed repeatedly with cold (4 °C) PBS solution (Szabo-Scandic, Vienna, Austria) before the amniotic membrane (AM) was carefully peeled off and separated from the chorion. The membrane was again washed repeatedly with cold PBS to remove blood remnants and then transferred to a 150 mm cell culture dish (Thermo Fisher Scientific, Waltham, MA, USA). The membranes were submerged in culture medium Dulbecco’s Modified Eagle’s Medium high-glucose, (DMEM-HG) supplemented with 10% fetal calf serum, (FCS), 1% L-glutamine, and 1% Pen/Strep) and cultured at 37 °C, 5% CO_2_ in a humidified atmosphere (CO_2_ incubator by Binder, Tuttlingen, Germany), until cell isolation on the following day.

For the isolation of hAMSCs, the membranes were washed twice with cold PBS and then dissected into pieces of about 2 × 3 cm^2^ each. Subsequently, the pieces were transferred to T75 cell culture flasks and incubated in 1-mg/mL collagenase solution (collagenase type I, CLS1, 280 U/mg, Worthington Biochemical Corporation, Lakewood, NJ, USA) at 37 °C and continuous shaking for 2 h. Afterwards, an equal volume of cold PBS was added, and the cell suspension was filtered through 100 and 22-µm cell strainers (Thermo Fisher Scientific, Waltham, MA, USA and Karlsruhe, Germany). The suspension was then centrifuged for 9 min at 400× *g* and 4 °C, and the cell pellet was resuspended in DMEM HG medium supplemented with 10% FCS, 1% L-glutamine, 1% Pen/Strep, 1 ng/mL basic fibroblast growth factor (bFGF, Peprotech, Rocky Hill, CT, USA), and 15 mM HEPES. Cells were seeded in T175 cell culture flasks (2.5 × 10^6^ cells/flask) and expanded for one passage. They were harvested at 70% confluency and cryopreserved at −80 °C.

After thawing, hAMSC were expanded in expansion medium (DMEM low-glucose, 10% FCS, 1% L-glutamine, 1% Pen/Strep, and 1 ng/mL bFGF (Peprotech, Rocky Hill, CT, USA) with 15 mM HEPES, passage 1) for 72 h and then split to 24-well plates for a pilot laser scan microscopy (LSM) analysis and a T75 flask, where they were further cultured in proliferation medium (DMEM/F-12, 10% FCS, 1% L-glutamine, 1% Pen/Strep, and 1 ng/mL bFGF (Peprotech, Rocky Hill, CT, USA) passage 2) for 72 h. The cells were split and cultivated in proliferation medium every 72 h, and passages 3 and 4 were used in the experiments.

### 2.3. Isolation of Human Bone Marrow-Derived Mesenchymal Stromal Cells

Human bone marrow mononuclear cells (hBMSCs) were purchased from Lonza (Basel, Switzerland), seeded on T75 tissue culture flasks and expanded in hBMSC culture medium consisting of DMEM- HG supplemented with 10% FCS, 1% Pen/Strep, 2 mM L-glutamine, and 1 ng/mL bFGF (Peprotech, Rocky Hill, CT, USA). Nonadherent bone marrow cells were removed during media changes. hBMSCs were routinely split upon reaching approx. 75% confluency. Trilineage differentiation potential and expression of mesenchymal surface antigens were confirmed (data not shown), and hBMSCs of passages 3, 4, and 5 were used in experiments.

### 2.4. Experimental Design

The effects of DPI on the mitochondrial membrane potential and cellular ROS levels were examined in undifferentiated hAMSCs, undifferentiated hBMSCs, and in hBMSCs after osteogenic induction for 14 days (osteoblast-like cells) in DMEM-HG supplemented with 10% FCS, 1% Pen/Strep, 2 mM L-glutamine, 10 nM dexamethasone, 50 µM ascorbic acid-2-phosphate, and 10 mM ß-glycerophosphate. Additionally, mitochondrial respiratory parameters were monitored in MG-63 cells (Merck, Sigma-Aldrich, St. Louis, MO, USA). Prior to the start of the experiments, the cells were synchronized by FCS starvation for 24 h. Synchronization was not performed on MG-63 cells.

### 2.5. Mitochondrial Respiration

Permeabilization of the cytoplasmic membrane of MG-63 cells was achieved by exposing the cells to 12 μM digitonin for 10 min. Permeabilized MG-63 cells were treated with different concentrations of DPI (0.625 µM, 1.25 µM, 2.5 µM, 5 µM, 10 µM, and 100 µM) and 0.5% dimethyl sulfoxide (DMSO) as the control. The concentration of MitoTEMPO (20 μM) was chosen based on the prior publication, where it was used to prevent oxidative damage caused by excessive mitochondrial ROS production [21]. MG-63 cells were seeded with a density of 10,000 cells/cm^2^ in T175 flasks in DMEM-HG, supplemented with 10% FCS and 1% L-glutamine, (“cell culture medium”). After 3 days’ incubation at 37 °C, 5% CO_2_, and humidified atmosphere, the cells reached a confluency of 75%. The cells were detached with 2 mL trypsin/ ethylene diamine tetraacetic acid (EDTA) solution (10× concentration, 25% trypsin/EDTA and 75% PBS), centrifuged (218× *g*, 5 min), and counted with a hemocytometer (Neubauer type, Marienfeld, Lauda-Königshofen, Germany).

For the initial dose dependency, the cells were incubated for 30 min with different concentrations of DPI (0.625 µM, 1.25 µM, 2.5 µM, 5 µM, 10 µM, and 100 µM) directly in the measurement chambers of the high-resolution respirometer (Oroboros Instruments, Innsbruck, Austria). A time point of 30 min was selected to test the direct effects of DPI. Delayed effects, possibly mediated by other intracellular processes, were examined in human primary cells only with laser scan microscopy (LSM; see below).

Permeabilized MG-63 were preincubated in cell culture flasks with 20 µM MitoTEMPO for 15 min and, after washing with PBS, treated with DPI for 30 min. Then, the cells were detached with trypsin/EDTA (10 × concentration, 25% trypsin/75% PBS), centrifuged (218× *g*, 5 min), counted with a hemocytometer (Neubauer type, Lauda-Königshofen, Germany), and examined with high-resolution respirometry (Oroboros Instruments, Innsbruck, Austria).

### 2.6. Mitochondrial Membrane Potential and Cellular ROS Measurement

Undifferentiated hBMSCs, hBMSCs after osteogenic induction, hAMSCs, and MG-63 cells were seeded in 24-well plates at a density of 10,000 cells/cm^2^ in 1 mL culture medium/well. The cells were cultured for 48 h until 70–80% confluency was reached and synchronized for 24 h by FCS starvation. Then, the cells were pretreated for 15 min in a cell culture medium supplemented with 20 μM MitoTEMPO or 0.05% DMSO (control). The medium was then changed to a proliferation medium supplemented with 10 µM DPI, 100 μM DPI, or 0.05% DMSO (control), and the cells were incubated for various times, ranging from 30 min or longer, up to 96 h. At the end of incubation, the cells were stained with fluorescent probes in Hank’s balanced salt solution according to the manufacturer’s instructions. Fluorescent dye tetramethylrhodamine methyl ester (TMRM, 50 nM, 30 min, 37 °C, ex/em 543/565–583 nm; Promokine, Heidelberg, Germany) was used to evaluate mitochondrial membrane potential, fluorescent dye CM-H2DCFDA (5-(and-6)-chloromethyl-2’,7’-dichlorodihydrofluorescein diacetate, acetyl ester, 10 μM, 30 min, 37 °C, ex/em 488/505–550 nm; Thermo Fischer, Waltham, MA, USA) was used for the detection of cellular ROS, and fluorescent dye CM-H2XRos, 5 μM, 30 min, 37 °C, ex/em 543/ 596–617 nm; Thermo Fischer, Waltham, MA, USA) was used for the detection of mitochondrial ROS. LSM imaging was performed with an inverted confocal microscope (LSM 510, Zeiss, Oberkochen, Germany) and 10× objective. Image analysis was performed with ZEN 2009 (version 6.0.303; Carl Zeiss, Oberkochen, Germany) based on the region of interest selection consisting of multiple cells. The mean fluorescence magnitude obtained from single cells was determined after setting threshold at 2/255 in order to subtract the background signal.

### 2.7. Measurement of Mitochondrial Respiration

Mitochondrial respiratory parameters were monitored using high-resolution respirometry (Oxygraph-2k, Oroboros Instruments, Innsbruck, Austria). MG-63 cells (2 × 10^6^/mL) were incubated in a buffer containing 115 mM KCl, 5 mM KH_2_PO_4_, 20 mM Tris-HCl, 0.5 mM EDTA, and 5 mg/mL fatty acid-free bovine serum albumin (pH 7.4, 37 °C). To assess Complex I- and Complex II-linked respiration, cells were permeabilized with digitonin (12 µM). State 2 respiration of Complex I was stimulated by the addition of 5 mM glutamate/5 mM malate. Transition to State 3 respiration was induced by the addition of adenosine diphosphate (ADP, 1 mM). Complex II-linked State 3 respiration was then stimulated with 10 mM succinate after the addition of Complex I inhibitor rotenone (1 µM). Maximum respiratory capacity was measured by the titration of carbonyl cyanide-4-(trifluoromethoxy)phenylhydrazone (FCCP) in steps of 0.5 µM. Respiration rates were obtained by calculating the negative time derivative of the measured oxygen concentration and subtraction of non-mitochondrial oxygen consumption (myxothiazol 10 µM).

### 2.8. Statistical Analysis

Prior to statistical analysis, the data was transformed to fraction of the control group, with the respective control group mean taken as 100%. The outliers were identified with the robust regression and outlier removal (ROUT) method (5% threshold), and ordinary ANOVA was performed on the whole dataset, with additional multiple comparisons of individual means against the mean of a control group using Fisher’s Least Significant Difference (LSD) test as the post-hoc test. All evaluations were performed using GraphPad Prism (GraphPad Software, San Diego, CA, USA, Version 8.0.1). For laser scanning microscopy experiments, the number of independent samples (*n*) is indicated as number of analyzed regions of interest, which comprise multiple adjacent cells with nonpathological morphology. For the measurement of mitochondrial respiration, the *n* number represents experiments with cells seeded on independent days. Data are presented as mean + SD. The significance level was set at 0.05 and is indicated as * *p* < 0.05, ** *p* < 0.01, *** *p* < 0.001, and **** *p* < 0.0001 compared to the control.

## 3. Results

**Figure 1:**
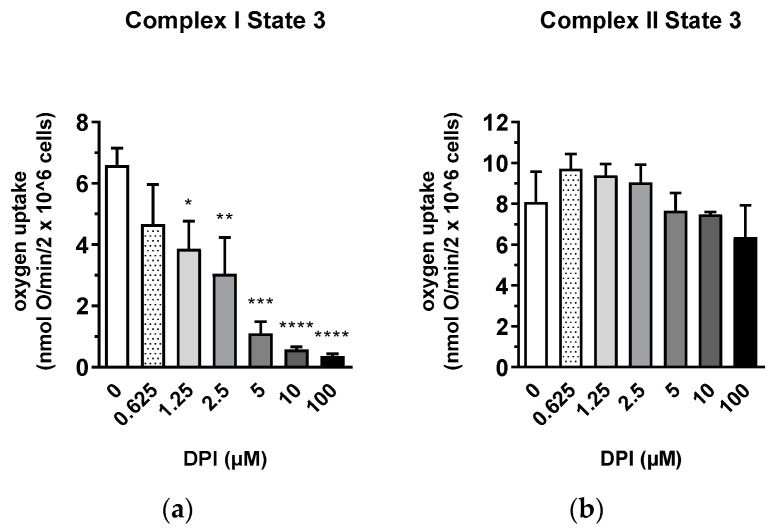
Inhibitory effect of diphenyleneiodonium chloride (DPI) on Complex I mitochondrial function in permeabilized MG-63 cells. (**a**) Effects of different concentrations of DPI on Complex I-linked State 3. * *p* < 0.05, ** *p* < 0.01, *** *p* < 0.001, and **** *p* < 0.0001 compared to the control. (**b**) Effects of different concentrations of DPI on Complex II-linked State 3. Data is represented as means + SD (error bars) (*n* = 3). One-way ANOVA followed by multiple comparison post-hoc Fisher’s LSD test.

The interaction between DPI and the mitochondria was first examined in permeabilized osteosarcoma cell line MG-63 to define the conditions for experiments with primary human cells, which were available in limited portions (Figure 1). Permeabilization allowed the testing of specific segments of the respiratory chain under conditions of substrate saturation (glutamate/malate or succinate). After 30 min of treatment, mitochondrial respiration with glutamate/malate (substrates of complex I) decreased with increasing DPI concentrations (0.625–100 M), reaching its minimum at approx. 5 µM DPI (Figure 1a). However, already at 0.625 µM DPI, we observed a strong inhibition of State 3 respiration linked to ATP synthesis. The recorded MG-63 mitochondrial respiration dataset is available in supplementary material (Figure S1). We showed that, although DPI inhibits the electron flow through Complex I, the total capacity of the respiratory chain determined in the presence of succinate and FCCP did not change in all ranges of DPI concentrations (Figure S1b).

In contrast, an increase in the rate of respiration was observed with succinate (a substrate of Complex II) after the treatment with DPI up to 10 µM and slightly dropped only upon treatment with 100 µM DPI (Figure 1b). We considered that the insufficiency of Complex I in MG-63 cells is compensated by Complex II in a wide range of DPI concentrations. Thus, for the following experiments, we selected 10 µM DPI as the concentration where the inhibition of Complex I can be fully compensated by electron flow via Complex II and 100 µM DPI as the critical concentration where mitochondrial dysfunction was expected to occur.

**Figure 2:**
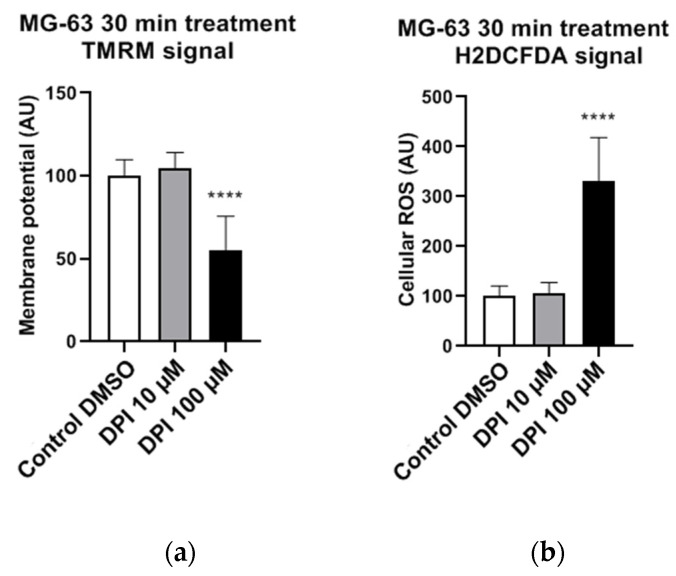
Effects of 30 min 10 µM and 100 µM diphenyleneiodonium chloride (DPI) in nonpermeabilized MG-63 cells, inducing a drop in mitochondrial membrane potential and a burst of reactive oxygen species (ROS) production on higher concentrations. The data is displayed in arbitrary units (AU). (**a**) Effects of 10 µM and 100 µM DPI on mitochondrial membrane potential. (**b**) Effects of 10 µM and 100 µM DPI on cellular ROS levels. Data is represented as means + SD (error bars) (*n* = 24; each point was obtained by analyzing 2–4 single cells. **** *p* < 0.0001; one-way ANOVA followed by multiple comparison post-hoc Fisher’s LSD test.).

The experiments with permeabilized MG-63 cells were performed under conditions of substrate saturation (glutamate/malate and succinate). That is not necessarily the case in living cells, where insufficient levels of intracellular succinate may induce a decrease in membrane potential if the electron transfer through Complex I is inhibited by DPI. To test this assumption, we determined the mitochondrial membrane potential in nonpermeabilized MG-63 cells in the presence of 10 and 100 µM DPI (Figure 2). We observed that 10 µM DPI did not influence the membrane potential, while 100 µM DPI significantly decreased the membrane potential (Figure 2a) and substantially elevated the levels of cellular ROS (Figure 2b).

**Figure 3:**
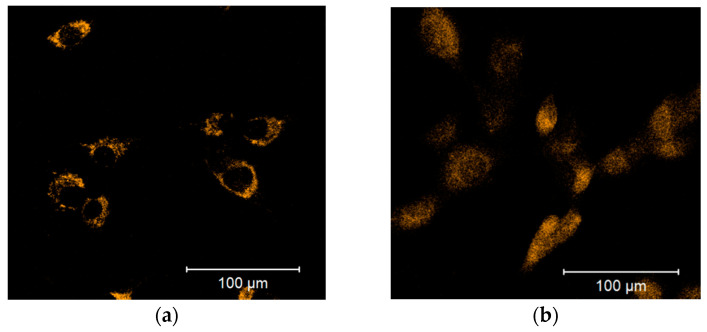
Release of the mitochondrial ROS-sensitive CM-H2XRos fluorescent probe to the cytoplasm is induced by a 100 µM diphenyleneiodonium chloride (DPI) treatment of MG-63 cells. (**a**) Control group treated with 0.5% vol. DMSO. (**b**) Group exposed to a 100 µM DPI concentration. The images were taken with a Zeiss LSM 510 microscope, 10× objective, 5 μM CM-H2XRos probe concentration.

**Figure 4:**
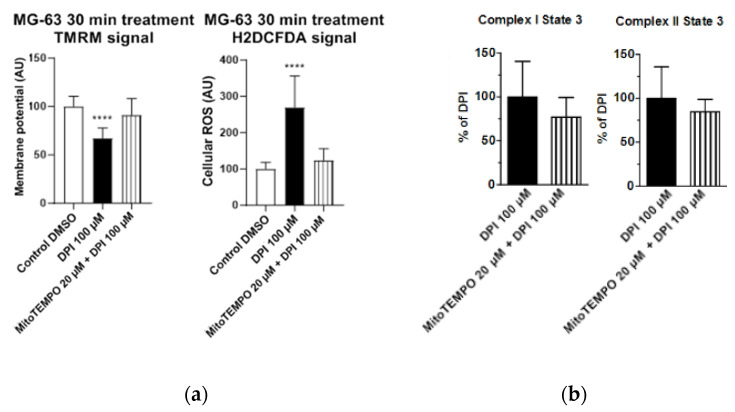
Effects of 20 μM MitoTEMPO pretreatment on MG-63 cells exposed to 100 µM DPI. (**a**) Mitochondrial membrane potential and cellular ROS levels estimated with laser scan microscopy (LSM). MitoTEMPO is capable of preventing a decrease of the mitochondrial membrane potential and excessive generation of ROS. The images were taken with a Zeiss LSM 510 microscope, 10x objective, 50 nM tetramethylrhodamine methyl ester (TMRM) and 10 µMH2DCFDA probe concentration. Data is represented as means + SD (error bars) (*n* = 24; each point was obtained by analyzing 2–4 single cells. **** *p* < 0.0001; one-way ANOVA followed by multiple comparison post-hoc Fisher’s LSD test. (**b**) Cellular respiration measurements of Complex I and Complex II State 3 MitoTEMPO does not cause recovery of mitochondrial respiration. Means + SD, *n* = 4.

Cellular ROS may come from different sources, including mitochondria. To examine whether the observed increase in cytoplasmic ROS originated from the mitochondria, we stained cells with a mitochondria-specific ROS-sensitive fluorescent probe and treated the cells for 30 min with 100 µM DPI (Figure 3). We observed that the ROS-sensitive fluorescent probe accumulated in the intact mitochondria of the control group (Figure 3a), whereas, in response to the DPI treatment, the fluorescent probe was released in the cytoplasm (Figure 3b), thus suggesting a mitochondrial origin of the ROS. Additionally, we stained the cells with CM-H2XRos together with a nuclear dye (NucSpot^®^ 650), and we did not find staining by CM-H2XRos in the nuclei of the control cells (Figure S2). To further prove the mitochondrial origin of cytoplasmic ROS, we tested whether the increase in ROS can be reduced by mitochondria-targeted antioxidant MitoTEMPO. If an excessive generation of ROS contributes to mitochondrial dysfunction, the treatment with MitoTEMPO should prevent a drop in membrane potential as well. Indeed, the LSM analysis showed that the treatment of MG-63 with MitoTEMPO abolished the increase in cytoplasmic ROS levels in DPI-treated cells and prevented the drop in the mitochondrial membrane potential (Figure 4a). The experiment performed with permeabilized cells with an excess of substrate MitoTEMPO did not affect the mitochondrial respiration recorded in the presence of 100 µM DPI (Figure 4b).

Figure 4 demonstrates our observations made with LSM and high resolution respirometry; the full cellular respirometry datasets are available in Figure S3. The data presented above suggest that the generation of cellular ROS and membrane potential are driven by the succinate-mediated pathway, which is in line with a recent finding that, in human mesenchymal stem cells, succinate plays an important role in maintaining stem cell functions [22]. Furthermore, the data presented above show that the LSM analysis using a combination of TMRM and H2DCFDA probes to analyze the changes in membrane potential induced by DPI or another drug is sufficient for a general estimation of the cellular bioenergetic status and ROS metabolism. The levels of cytoplasmic ROS represent an important feature of ROS metabolism, particularly for ROS signaling [3,23]. To examine whether the permeabilization used for the detection of mitochondrial respiration influences the measurements with fluorescent probes, we repeated the experiments with permeabilized cells (Figure S4). Permeabilization increased the susceptibility of cells to 100 µM DPI, reducing strongly the amount of attached cells (Figure S5). The remaining cells had the same membrane potential as the control cells and cells treated with 10 µM DPI in contrast to nonpermeabilized cells, which showed a decrease of the membrane potential in response to 100 µM DPI (Figure 2a). We assume that this difference is due to a drastic increase in the intracellular glucose levels, which additionally stimulated the electron flow through the respiratory chain. There was no difference in ROS levels in response to DPI treatments in nonpermeabilized (Figure 2b) and permeabilized cells (Figure S4b).

Determination of mitochondrial respiration in permeabilized cells showed that the inhibition of Complex I by DPI can be compensated by the electron supply from the succinate to Complex II. However, these measurements were performed under conditions of excessive succinate availability from the medium. Nevertheless, the intracellular amounts of succinate may not be sufficient to compensate the inhibition of Complex I. To prove this, we inhibited Complex I with rotenone and determined its effects on the mitochondrial membrane potential and cellular ROS levels with and without DPI.

Interestingly, a low concentration of DPI combined with rotenone caused mitochondrial membrane hyperpolarization (Figure S6a), similar to our observations with the effects of DPI and rotenone in mitochondrial respiration measurements (Figure 1b). The cellular ROS levels were not affected in the 10 µM DPI treatment group and strongly increased in the 100 µM DPI group, similarly to the experiment results without rotenone (Figure 2b). This suggests that the increase of cellular ROS levels is not due to the reverse electron flow from the succinate.

Therefore, in the following set of experiments, we used these parameters to estimate the bioenergetics status and ROS metabolism in hAMSCs and undifferentiated hBMSCs, as well as their osteogenic-induced progeny, osteoblast-like cells. 

### 3.1. 30-Minute Time Point

#### 3.1.1. hAMSC

**Figure 5:**
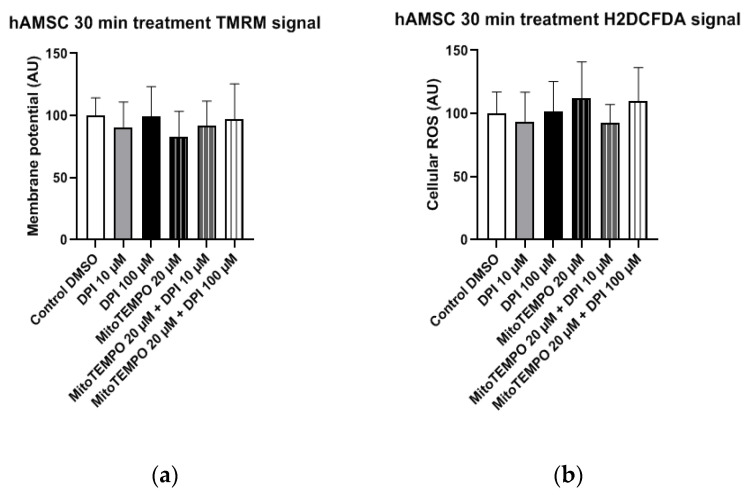
Mitochondrial membrane potential and cellular ROS levels 30 min after the treatment of human amniotic membrane mesenchymal stromal cells (hAMSCs) with diphenyleneiodonium chloride (DPI) and MitoTEMPO. (**a**) Changes in the mitochondrial membrane potential are not present. (**b**) Changes of the cellular ROS levels are not present. Data is represented as means + SD (error bars) (*n* = 24; each point was obtained by analyzing 2–4 single cells. *p* > 0.05; one-way ANOVA followed by multiple comparison post-hoc Fisher’s LSD test. There was no significant differences.

No statistically significant changes in mitochondrial membrane potential (Figure 5a) and cellular ROS levels (Figure 5b) were observed in hAMSC after the treatments with DPI (10 µM and 100 µM), suggesting the overall robustness of hAMSC in response towards all presented treatments.

#### 3.1.2. hBMSC

**Figure 6:**
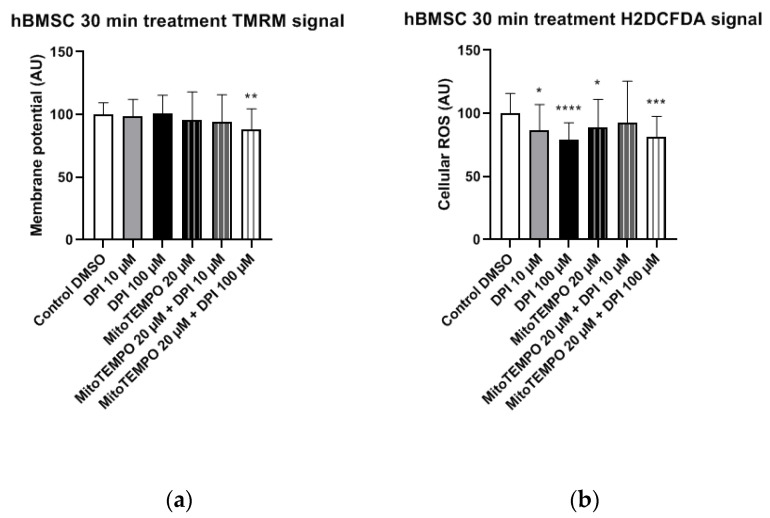
Mitochondrial membrane potential and cellular ROS levels 30 min after the treatment of hBMSCs with diphenyleneiodonium chloride (DPI) and MitoTEMPO. (**a**) Changes in the mitochondrial membrane potential. (**b**) Changes of the cellular ROS levels. Data is represented as means + SD (error bars) (*n* = 36; each point was obtained by analyzing 2–4 single cells. * *p* < 0.05, ** *p* < 0.01, *** *p* < 0.001, and **** *p* < 0.0001; one-way ANOVA followed by multiple comparison post-hoc Fisher’s LSD test.

In hBMSC, a combined treatment with 20 μM MitoTEMPO and 100 μM DPI substantially reduced the membrane potential in hBMSCs, whereas no significant differences were observed with the single MitoTEMPO and DPI treatment groups, even with the 100 µMDPI treatment (Figure 6a).

The treatments of hBMSCs with 10 μM and 100 μM DPI significantly reduced the cellular ROS levels (Figure 6b). An additional treatment with 20 μM MitoTEMPO did not affect the ROS levels compared to 10 µM DPI alone (Figure 6b). Taken together, the ROS level reduction in response to DPI and the absence of a MitoTEMPO effect implies that predominant target of DPI is NOX, not the mitochondria.

#### 3.1.3. Osteoblast-Like Cells

**Figure 7:**
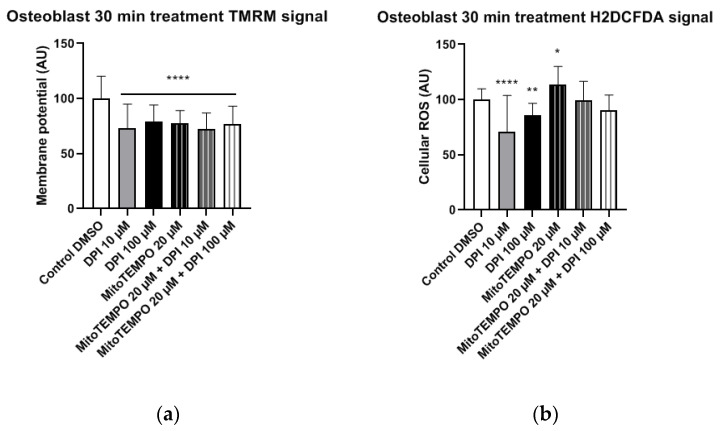
Mitochondrial membrane potential and cellular ROS levels 30 min after treatment of osteogenic-induced hBMSCs (osteoblast-like cells) with diphenyleneiodonium chloride (DPI) and MitoTEMPO. (**a**) Changes in the mitochondrial membrane potential. (**b**) Changes of the cellular ROS levels due to nicotinamide adenine dinucleotide phosphate oxidase (NOX) inhibition at high and low concentrations of DPI. Data is represented as means + SD (error bars) (*n* = 24; each point was obtained by analyzing 2–4 single cells. * *p* < 0.05, ** *p* < 0.01, and **** *p* < 0.0001; one-way ANOVA followed by multiple comparison post-hoc Fisher’s LSD test.

In osteoblast-like cells, all treatments resulted in significantly reduced mitochondrial membrane potential compared to the control group (Figure 7a). Furthermore, treatments with 10 μM and 100 μM DPI significantly reduced the cellular ROS levels compared to the control group (Figure 7b). This reveals that, similarly to undifferentiated hBMSCs, DPI showed inhibitory properties in predifferentiated cells. The treatment with 20 μM MitoTEMPO caused an increase of ROS compared to the control group, whereas the combined treatments exhibited no differences in the control group in these cells (Figure 7b).

### 3.2. Three-Hour Time Point

#### 3.2.1. hAMSCs

**Figure 8:**
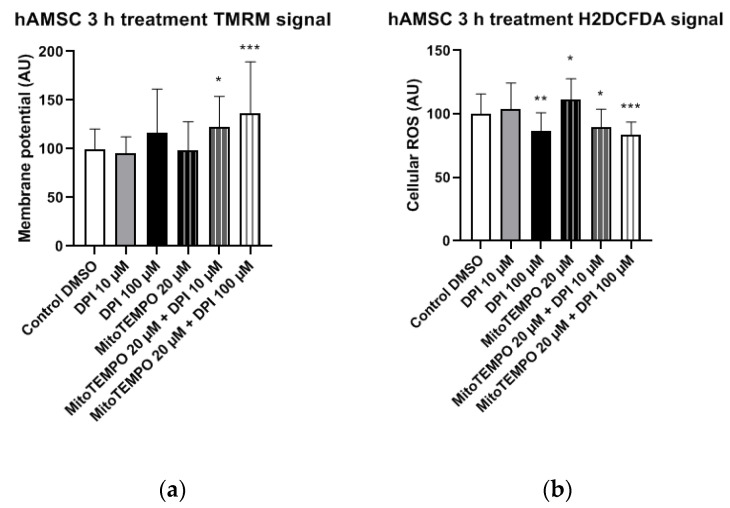
Mitochondrial membrane potential and cellular ROS levels 3 h after the treatment of hAMSCs with diphenyleneiodonium chloride (DPI) and MitoTEMPO. (**a**) Changes in mitochondrial membrane potential. (**b**) Changes of cellular ROS levels; high concentrations of DPI induce an increase of the ROS via the mitochondrial effects. Data is represented as means + SD (error bars) (*n* = 23; each point was obtained by analyzing 2–4 single cells. * *p* < 0.05, ** *p* < 0.01, and *** *p* < 0.001; one-way ANOVA followed by multiple comparison post-hoc Fisher’s LSD test.

In hAMSCs, we observed a significant increase in the mitochondrial membrane potential in the combined treatment groups with 20 µM MitoTEMPO and either 10 µM or 100 µMDPI (Figure 8a). A similar trend of increased mitochondrial membrane potential was observed in the 100 µM DPI group, but the difference to the control group was not statistically significant (Figure 8a). The treatment of hAMSCs with 100 µM DPI and combined treatments of hAMSCs with 20 µM MitoTEMPO and 10 µMor 100 µM DPI resulted in significantly decreased cellular ROS levels compared to the control group (Figure 8b). This could imply a presence of crosstalk between the mitochondria and NOX in hAMSCs, as the decrease in ROS levels was accompanied by an increase in the mitochondrial membrane potential. Paradoxically, the treatment with 20 µM MitoTEMPO caused a significant increase in cellular ROS levels compared to the control group (Figure 8b).

#### 3.2.2. hBMSCs

**Figure 9:**
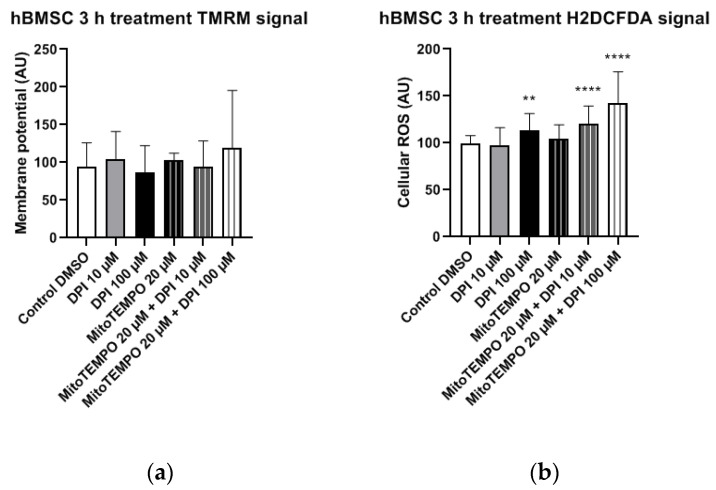
Mitochondrial membrane potential and cellular ROS levels 3 h after treatment of hBMSCs with diphenyleneiodonium chloride (DPI) and MitoTEMPO. (**a**) Changes in the mitochondrial membrane potential. (**b**) Changes of cellular ROS levels; the increase is caused by the mitochondrial effects of DPI. Data is represented as means + SD (error bars) (*n* = 36; each point was obtained by analyzing 2–4 single cells. ** *p* < 0.01, and **** *p* < 0.0001; one-way ANOVA followed by multiple comparison post-hoc Fisher’s LSD test.

In hBMSCs, no statistically significant changes in the mitochondrial membrane potential were observed in any of the treatment groups compared to the control group (Figure 9a). The treatment of hBMSCs with 100 µM DPI and combined treatments with 20 µM MitoTEMPO and 10 µM or 100 µM DPI led to significant increases in the cellular ROS levels compared to the control group (Figure 9b). This suggests the onset of DPI effects on the mitochondria, which were not prevented but, rather, exacerbated in a combined treatment with MitoTEMPO and 100 µM DPI compared to the treatment with 100 µM DPI alone (Figure 9b).

We observed a relatively big variance in the 100 µM DPI plus 20 µM MitoTEMPO groups (Figure 8 and Figure 9). We assume the following reasons for that. In our experiments, the primary cells first underwent synchronization for 24 h. This implies that, 30 min after treatments, the cells were still synchronized; however, by three h, a portion of the cells already responded to the treatments changing their metabolic states, and another portion still did not. Eventually, all cells reached a steady state by 24 h. We presume that this is the reason for the higher variations at the three-h time point, including the combined treatment group with 100 µM DPI plus 20 µM MitoTEMPO.

#### 3.2.3. Osteoblast-Like Cells

**Figure 10:**
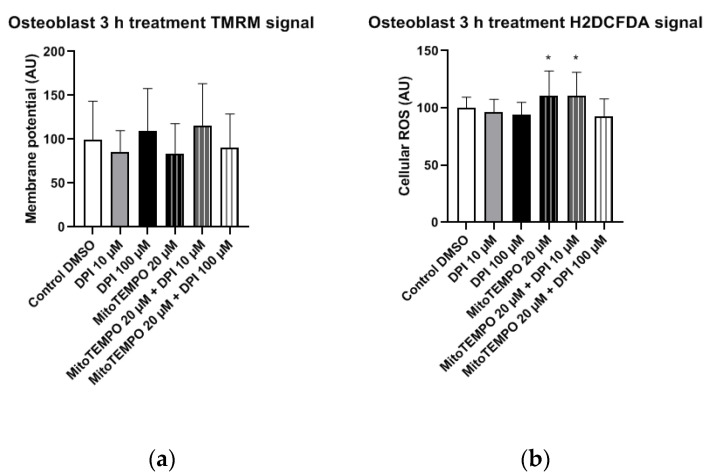
Mitochondrial membrane potential and cellular ROS levels 3 h after the treatment of osteogenic-induced hBMSCs (osteoblast-like cells) with diphenyleneiodonium chloride (DPI) and MitoTEMPO. (**a**) Changes in the mitochondrial membrane potential. (**b**) Changes of the cellular ROS levels. Data is represented as means + SD (error bars) (*n* = 24; each point was obtained by analyzing 2–4 single cells; cells used in these experiments were obtained from the differentiating passage 2 hBMSCs. * *p* < 0.05; one-way ANOVA followed by multiple comparison post-hoc Fisher’s LSD test.

In osteoblast-like cells, no statistically significant differences in mitochondrial membrane potential changes were observed in any of the treatment groups compared to the control group (Figure 10a). However, the treatment with 20 µM MitoTEMPO, as well as the combined treatment with 20 µM MitoTEMPO and 10 µM DPI caused a statistically significant increase in the cellular ROS levels compared to the control group (Figure 10b).

### 3.3. 24-Hour Time Point

#### 3.3.1. hAMSCs

**Figure 11:**
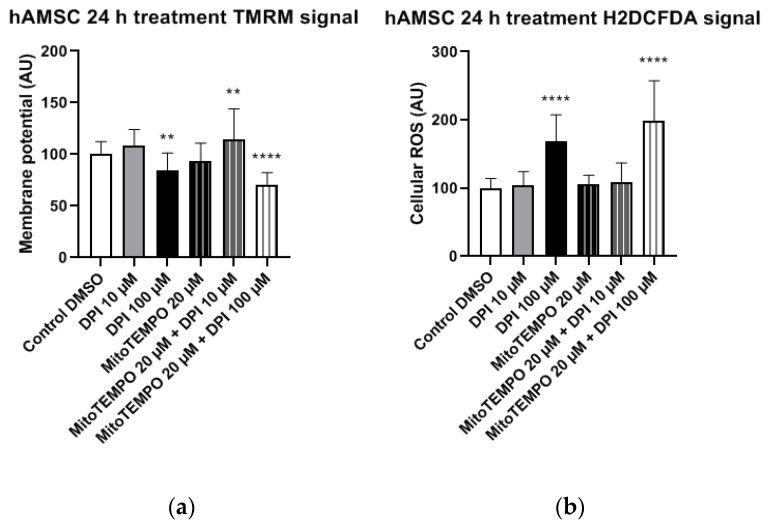
Mitochondrial membrane potential and cellular ROS levels 24 h after the treatment of hAMSCs with diphenyleneiodonium chloride (DPI) and MitoTEMPO. (**a**) Changes in the mitochondrial membrane potential. (**b**) Changes of the cellular ROS levels, an increase caused by a high DPI. Data is represented as means + SD (error bars) (*n* = 23; each point was obtained by analyzing 2–4 single cells. ** *p* < 0.01, and **** *p* < 0.0001; one-way ANOVA followed by multiple comparison post-hoc Fisher’s LSD test.

In hAMSCs, a significant decrease in the mitochondrial membrane potential was observed after the treatment with 100 µM DPI and the combined treatment with 20 µM MitoTEMPO and 100 µM DPI (Figure 11a), which showed a direct inhibitory effect on the mitochondria. However, an increase in the mitochondrial membrane potential was observed after the combined treatment with 20 µM MitoTEMPO and 10 µM DPI (Figure 11a). The treatment with 100 µM DPI and combined treatment with 20 µM MitoTEMPO and 100 µM DPI induced a significant increase in the cellular ROS levels compared to the control group (Figure 11b). Thus, the 20 µM MitoTEMPO and 100 µM DPI treatment group exhibited a simultaneous decrease in mitochondrial membrane potential with an increase in the cellular ROS, suggesting a direct inhibitory effect of high concentrations of DPI on the mitochondria.

#### 3.3.2. hBMSCs

**Figure 12:**
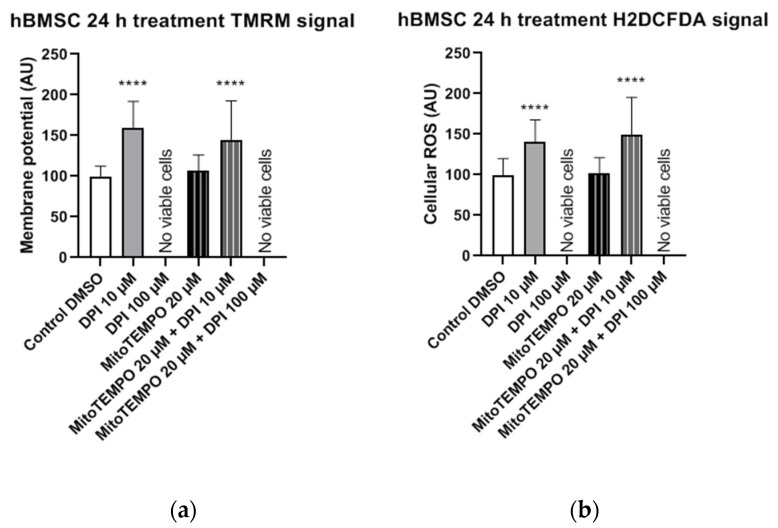
Mitochondrial membrane potential and cellular ROS levels 24 h after the treatment of hBMSCs with diphenyleneiodonium chloride (DPI) and MitoTEMPO. (**a**) Changes in the mitochondrial membrane potential. (**b**) Changes of the cellular ROS levels. Data is represented as means + SD (error bars) (*n* = 36; each point was obtained by analyzing 2–4 single cells. **** *p* < 0.0001; one-way ANOVA followed by multiple comparison post-hoc Fisher’s LSD test.

**Figure 13:**
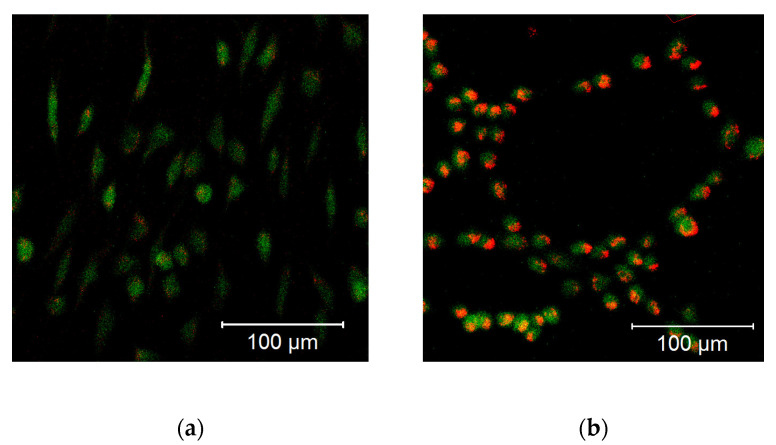
Morphological changes of the hBMSCs (passage 3) in response to the 10 µM DPI 24-h treatment, mitochondrial membrane potential-sensitive TMRM probe, and cellular ROS-sensitive H2DCFDA probe. (**a**) Control group treated with 0.5% vol. DMSO. (**b**) Group treated with 10 µM DPI. The images were taken with a Zeiss LSM 510 microscope, 10× objective, 50 nM TMRM (red signal) or 10 µM (green signal) H2DCFDA probe concentration.

In hBMSCs, both the mitochondrial membrane potential (Figure 12a) and cellular ROS levels (Figure 12b) showed a strong increase after the treatment with 10 µM DPI and the combined treatment with 20 µM MitoTEMPO and 10 µM DPI. However, no viable cells could be observed after the 24-h treatment with either 100 µM DPI alone or in combination with 20 µM MitoTEMPO (Figure 12). Additionally, morphological changes of the hBMSC shape compared to the control group were observed after the 10 µM DPI treatment (Figure 13). The cells appeared in a compacted form, building up round structures while exhibiting elevated mitochondrial membrane potential (Figure 12).

This suggests that a treatment with low DPI concentrations affects the mitochondrial activity of hBMSCs. Similar changes in the morphology were observed in after the treatment with 20 µM MitoTEMPO (data not shown). Taking into consideration the 3-h time point results, our data imply that, in hBMSCs, at a certain point after the DPI treatments, the scales are tipped from ROS reduction by 10 µM DPI via NOX inhibition (30-min time point) to ROS production by mitochondria (3 h and 24 h). However, hBMSCs treated with 100 µM DPI showed an increase of cellular ROS levels after 3 h, which eventually led to death after 24 h.

#### 3.3.3. Osteoblast-Like Cells

**Figure 14:**
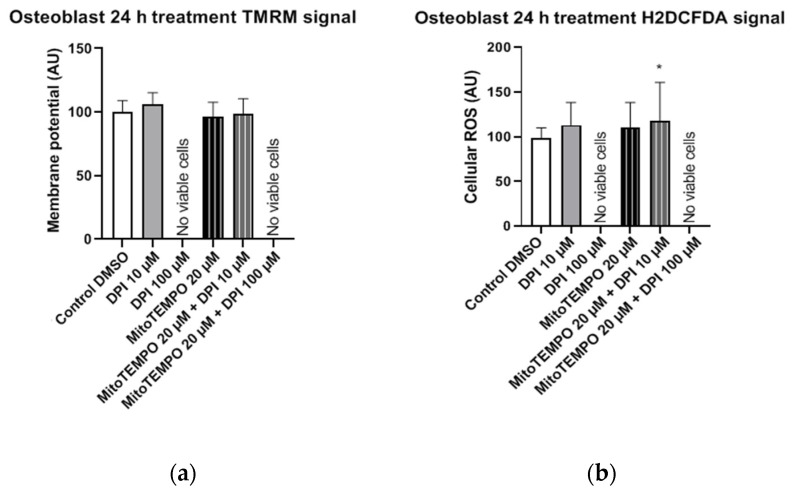
Mitochondrial membrane potential and cellular ROS levels 24 h after the treatment of osteogenic-induced hBMSCs (osteoblast-like cells) with diphenyleneiodonium chloride (DPI) and MitoTEMPO. (**a**) Changes in the mitochondrial membrane potential. (**b**) Changes of the cellular ROS levels. Data is represented as means + SD (error bars) (*n* = 24; each point was obtained by analyzing 2–4 single cells; the cells used in these experiments were obtained from the differentiating passage 2 hBMSCs. * *p* < 0.05; one-way ANOVA followed by multiple comparison post-hoc Fisher’s LSD test.

In osteoblast-like cells, single and combined treatments with 10 µM DPI and 20 µM MitoTEMPO resulted in cell survival but no statistically significant alterations in mitochondrial membrane potential compared to the control group (Figure 14a). In contrast, a statistically significant increase in cellular ROS levels was detected after the combined treatment with 20 µM MitoTEMPO and 10 µM DPI (Figure 13b). Overall, this implies an increased robustness of osteoblast-like cells compared to their undifferentiated hBMSC counterparts, since no ROS increase was found after the treatment with 10 µM DPI after 24 h. However, similarly to hBMSCs, no viable osteoblast-like cells were detected after the single or combined treatments with 100 µM DPI and 20 µM MitoTEMPO (Figure 14). To better visualize the similarities within the cell types examined in this study, we applied a Ward’s cluster analysis (Figure S7). We analyzed a total of 10 predictors of all H2DCFDA and TMRM groups. Cells connected closer to 0 on the x-axis were more similar to each other, e.g., BMSC 3 h and hAMSC at 24 h were more similar than the ROS at 24 h and BMSC at 24 h.

## 4. Discussion

The main findings of this study highlight the differences in ROS turnover between MG-63 cells, hAMSCs, hBMSCs, and hBMSCs differentiated towards osteogenic lineage in vitro. The treatment with 100 µM DPI induced the inhibition of mitochondrial Complex I-linked respiration in MG-63 cells (Figure 1 and Figure 2), accompanied by a burst of mitochondrial ROS production, followed by the translocation of ROS into the cytoplasm (Figure 3). This translocation was accompanied by a decrease in mitochondrial membrane potential. Assuming that excessive ROS generation by the mitochondria may be the reason for the decreased membrane potential, we pretreated cells with mitochondria targeted ROS scavenger MitoTEMPO. Indeed, a drop in membrane potential was prevented by this antioxidant in MG-63 cells. In contrast to MG-63 cells, in hAMSCs, hBMSCs, and osteoblast-like cells derived from hBMSCs, 100 µM DPI did not induce excessive ROS production.

We examined and confirmed the potency of DPI to inhibit mitochondrial respiratory Complex I even at concentrations of 0.625 μM in MG-63 cells, while the Complex II activity remained unaffected. This suggests the presence of a compensatory mechanism, which may explain the lack of acute response towards the 10 µM DPI treatment. Indeed, 10 µM of DPI nearly fully inhibited Complex I but did not influence the membrane potential determined by the membrane potential sensitive fluorescent dye TMRM. In contrast, 100 µM DPI, which already partially affected Complex II (Figure 1) induced a drop in the mitochondrial membrane potential and elevated the ROS levels (Figure 4). Here, we should note that, in the experiments with mitochondrial respiration, the cells were permeabilized and had access to saturating substrate concentrations from the culture medium. In contrast, in the nonpermeabilized cells, the concentrations of substrate can limit the mitochondrial function, and, consequently, the effects of the DPI treatment can be stronger. Furthermore, our experiments with the MG-63 cells suggested that the determination of the mitochondrial membrane potential and the levels of cytoplasmic ROS with fluorescent probes, in combination with the effects of the mitochondria-targeted antioxidant MitoTEMPO, may bring key information about the ROS metabolism in various cell types. We considered that both NOX and mitochondria can contribute to the cellular pool of ROS and affect the mitochondrial function. In this sense, the application of MitoTEMPO allowed us to dissect the specific contribution of mitochondrial ROS. This strategy was applied for hAMSCs, hBMSCs, and differentiated hBMSCs.

Compared to MG-63 cells, hAMSCs of the reflected amniotic region are characterized by low mitochondrial activity [24] and possibly rely mostly on glycolytic pathways. This implies that hAMSC ROS metabolism is more likely to be influenced by NADPH oxidase (NOX) activity rather than the mitochondria. The 30-min treatments with DPI alone or in combination with MitoTEMPO did not result in any alterations in the hAMSC mitochondrial membrane potential and cellular ROS levels compared to the controls (Figure 5). After the three-h treatment, we observed a decrease in cellular ROS in groups treated with high concentrations of DPI, accompanied by an increase in the mitochondrial membrane potential. This effect was slightly more pronounced in the presence of MitoTEMPO. However, after 24 h of exposure to 100 µM DPI alone or in combination with MitoTEMPO, we observed an increase in cellular ROS, accompanied by a decrease in mitochondrial membrane potential. Together, these data suggest that the ROS metabolic pathways in hAMSCs are predominantly determined by NOX rather than by mitochondrial ROS, and high concentrations of DPI did not reduce substantially the numbers of cells by 24 h.

In experiments with hBMSCs, after 30 min, most single and combined treatments with DPI and MitoTEMPO reduced the levels of cytoplasmic ROS. An accompanying decrease of the mitochondrial membrane potential was found only in the combined treatment group with 20 µM MitoTEMPO and 100 µM DPI (Figure 6); 10 µM and 100 µM DPI did not affect the membrane potential but decreased the levels of cytoplasmic ROS. As the highest effects were observed with 100 µM DPI either with or without MitoTEMPO, our data suggests the involvement of NOX inhibition, similar to what we observed in hAMSCs after three h. After three h, the treatment of hBMSCs with 100 µM DPI either alone or in combination with MitoTEMPO elevated the cytoplasmic ROS production, an effect similar to what was observed at 24 h with the hAMSCs. However, the three-h treatment with DPI did not influence membrane potential. Surprisingly, we also observed an increase in the levels of cellular ROS upon the combined treatment with both 10 µM DPI and MitoTEMPO. We cannot explain this phenomenon; however, it demonstrates that MitoTEMPO does not exert a protective effect in these cells. After 24 h of hBMSC treatment with 100 µM DPI either alone or in combination with MitoTEMPO, more than 90% of the cells were dead (Figure 12). This shows that 100 µM DPI is toxic for hBMSCs, although it was not toxic for hAMSCs. Interestingly, the hBMSCs treated with 10 µM DPI exhibited an increase in both cellular ROS and mitochondrial membrane potential, accompanied by drastic changes in the morphology (Figure 13). These changes indirectly suggested that 10 µM DPI might affect the intracellular signaling processes potentially associated with cellular differentiation.

Consequently, our results show that the ROS turnover in hBMSCs is initially more influenced by NOX activity rather than by the mitochondria, with the mitochondria-related effects beginning to contribute after 3 h. Taken together, in both the hAMSCs as well as the hBMSCs, the initial response to the DPI treatment was mediated by NOX, while the mitochondrial response occurred after 24 h in hAMSCs and 3 h in hBMSCs.

We next examined the osteoblast-like cells differentiated from hBMSCs 30 min after DPI treatment (Figure 7). We observed a decrease in the cytoplasmic ROS levels in response to either 10 µM or 100 µM DPI. This response was not accompanied by an increase in the membrane potential. After three h, we did not see any changes in osteoblast-like cell membrane potentials, but we did observe a slight elevation of the cytoplasmic ROS by 20 µM MitoTEMPO alone or in combination with DPI (Figure 10). We do not have a reasonable explanation for this fact, perhaps other than the NOX or mitochondria source of ROS appears at this time point. After 24 h, there were no vital osteoblast-like cells upon the treatment with 100 µM DPI, similar with the hBMSCs (Figure 14). The mitochondrial membrane potential was not affected in all groups that survived the treatment; the levels of cellular ROS were increased only in the 20 µM MitoTEMPO combined with 10 µM DPI group. While the mode of action of MitoTEMPO as an antioxidant is well-defined in immortalized cell lines, here, we observed an opposite effect. This poses an open question regarding the other possible effects of MitoTEMPO in primary cells.

Our results suggest that the ROS metabolism in osteogenic predifferentiated hBMSCs is more dependent on mitochondrial ROS than on not differentiated BMSCs. This is in-line with the previous findings that undifferentiated cells are more dependent on the anerobic energy metabolism [25]. Consequently, DPI affects undifferentiated cells predominantly by inhibiting NOX, while, in predifferentiated cells, the mitochondrial effects of DPI become more pronounced. Consequently, our approach for the simultaneous determination of the mitochondrial membrane potential and cytoplasmic ROS can distinguish specific differences in the ROS metabolism in different types of mesenchymal stromal cells at different phases of their proliferation and differentiation. A Ward’s cluster analysis (supplementary material, Figure S3) could further enhance the understanding of cell similarities after different treatments (with DPI) at different stages of differentiation.

## 5. Conclusions

Our study demonstrates critical differences in ROS metabolism between the osteoblastoma cell line (MG-63), osteoblast-like cells induced from hBMSCs, and undifferentiated hBMSCs and hAMSCs. These differences were uncovered by the examination of the cell responses to treatments with DPI and MitoTEMPO. Our data suggest that the mechanism of ROS generation and the levels of intracellular ROS predominantly depend on the type of energy metabolism used by the cell. Thus, in cell types with glycolysis, DPI predominantly interacts with NOX, while the mitochondria remain unaffected. In contrast, in cells with aerobic energy metabolism, the mitochondria become an additional target for DPI. As a result, cells relying more on aerobic metabolism such as MG-63 or osteoblast-like cells are more sensitive to the toxic effects of DPI, while cells predominantly living from glycolysis, such as hAMSCs, are more resistant to the toxic effects of DPI. In summary, our data suggest that undifferentiated cells rather than differentiated parenchymal cells should be considered as potential targets for DPI.

## Data Availability

Data available on request due to ethical reasons.

## Supplementary Materials

The following are available online at https://www.mdpi.com/1999-4923/13/1/10/s1: Figure S1. Effects of diphenyleneiodonium chloride (DPI) on the mitochondrial function in MG-63 cells, Figure S2. MG-63 cell nuclear and mitochondrial ROS staining in the control cells, Figure S3. Combined effects of diphenyleneiodonium (DPI) and MitoTEMPO in permeabilized, Figure S4. Mitochondrial membrane potential and cellular ROS levels in permeabilized MG-63 cells, Figure S5. Response of permeabilized and nonpermeabilized MG-63 cells to 100 μM DPI, Figure S6. Mitochondrial membrane potential and cellular ROS levels in MG-63 cells exposed to DPI and 5 µM rotenone, Figure S7. Hierarchical cluster analysis of the hAMSCs, hBMSCs, and osteoblast similarity dendrogram.

## References

[1] Dikalov S. (2011). Cross talk between mitochondria and NADPH oxidases. Free Radic. Biol. Med..

[2] Daiber A. (2010). Redox signaling (cross-talk) from and to mitochondria involves mitochondrial pores and reactive oxygen species. Biochim. Biophys. Acta (BBA)-Bioenerg..

[3] Weidinger A., Kozlov A.V. (2015). Biological activities of reactive oxygen and nitrogen species: Oxidative stress versus signal transduction. Biomolecules.

[4] Wang Q., Qian L., Chen S.H., Chu C.H., Wilson B., Oyarzabal E., Ali S., Robinson B., Rao D., Hong J.S. (2015). Post-treatment with an ultra-low dose of NADPH oxidase inhibitor diphenyleneiodonium attenuates disease progression in multiple Parkinson’s disease models. Brain.

[5] Nagel S., Genius J., Heiland S., Horstmann S., Gardner H., Wagner S. (2007). Diphenyleneiodonium and dimethylsulfoxide for treatment of reperfusion injury in cerebral ischemia of the rat. Brain Res..

[6] Nagel S., Hadley G., Pfleger K., Grond-Ginsbach C., Buchan A.M., Wagner S., Papadakis M. (2012). Suppression of the inflammatory response by diphenyleneiodonium after transient focal cerebral ischemia. J. Neurochem..

[7] Kuai Y., Liu H., Liu D., Liu Y., Sun Y., Xie J., Sun J., Fang Y., Pan H., Han W. (2020). An ultralow dose of the NADPH oxidase inhibitor diphenyleneiodonium (DPI) is an economical and effective therapeutic agent for the treatment of colitis-associated colorectal cancer. Theranostics.

[8] Buggisch M., Ateghang B., Ruhe C., Strobel C., Lange S., Wartenberg M., Sauer H. (2007). Stimulation of ES-cell-derived cardiomyogenesis and neonatal cardiac cell proliferation by reactive oxygen species and NADPH oxidase. J. Cell Sci..

[9] Ozsvari B., Bonuccelli G., Sanchez-Alvarez R., Foster R., Sotgia F., Lisanti M.P. (2017). Targeting flavin-containing enzymes eliminates cancer stem cells (CSCs), by inhibiting mitochondrial respiration: Vitamin B2 (Riboflavin) in cancer therapy. Aging (Albany. NY).

[10] Daiber A., Di Lisa F., Oelze M., Kröller-Schön S., Steven S., Schulz E., Münzel T. (2017). Crosstalk of mitochondria with NADPH oxidase via reactive oxygen and nitrogen species signalling and its role for vascular function. Br. J. Pharmacol..

[11] Silini A.R., Cargnoni A., Magatti M., Pianta S., Parolini O. (2015). The long path of human placenta, and its derivatives, in regenerative medicine. Front. Bioeng. Biotechnol..

[12] Caplan A.I. (1991). Mesenchymal stem cells. J. Orthop. Res..

[13] Kim J., Kang H.M., Kim H., Kim M.R., Kwon H.C., Gye M.C., Kang S.G., Yang H.S., You J. (2007). Ex vivo characteristics of human amniotic membrane-derived stem cells. Cloning Stem Cells.

[14] Sadler T. (2004). Langman’s Medical Embryology.

[15] Caplan A.I., Dennis J.E. (2006). Mesenchymal stem cells as trophic mediators. J. Cell. Biochem..

[16] Zupan J., Tang D., Oreffo R.O.C., Redl H., Marolt Presen D. (2020). Bone-Marrow-Derived Mesenchymal Stromal Cells: From Basic Biology to Applications in Bone Tissue Engineering and Bone Regeneration. Cell Engineering and Regeneration.

[17] Chen C.-T., Shih Y.-R.V., Kuo T.K., Lee O.K., Wei Y.-H. (2008). Coordinated Changes of Mitochondrial Biogenesis and Antioxidant Enzymes During Osteogenic Differentiation of Human Mesenchymal Stem Cells. Stem Cells.

[18] Chen C.T., Hsu S.H., Wei Y.H. (2010). Upregulation of mitochondrial function and antioxidant defense in the differentiation of stem cells. Biochim. Biophys. Acta (BBA)-Gen. Subj..

[19] Banerjee A., Weidinger A., Hofer M., Steinborn R., Lindenmair A., Hennerbichler-Lugscheider S., Eibl J., Redl H., Kozlov A.V., Wolbank S. (2015). Different metabolic activity in placental and reflected regions of the human amniotic membrane. Placenta.

[20] Hennerbichler S., Reichl B., Pleiner D., Gabriel C., Eibl J., Redl H. (2007). The influence of various storage conditions on cell viability in amniotic membrane. Cell Tissue Bank..

[21] Chang H.W., Wang H.R., Tang J.Y., Wang Y.Y., Farooqi A.A., Yen C.Y., Yuan S.S.F., Huang H.W. (2019). Manoalide preferentially provides antiproliferation of oral cancer cells by oxidative stress-mediated apoptosis and dna damage. Cancers.

[22] Ko S.H., Choi G.E., Oh J.Y., Lee H.J., Kim J.S., Chae C.W., Choi D., Han H.J. (2017). Succinate promotes stem cell migration through the GPR91-dependent regulation of DRP1-mediated mitochondrial fission. Sci. Rep..

[23] Kozlov A.V., Lancaster J.R., Meszaros A.T., Weidinger A. (2017). Mitochondria-meditated pathways of organ failure upon inflammation. Redox Biol..

[24] Banerjee A., Lindenmair A., Hennerbichler S., Steindorf P., Steinborn R., Kozlov A.V., Redl H., Wolbank S., Weidinger A. (2018). Cellular and Site-Specific Mitochondrial Characterization of Vital Human Amniotic Membrane. Cell Transpl..

[25] Ito K., Suda T. (2014). Metabolic requirements for the maintenance of self-renewing stem cells. Nat. Rev. Mol. Cell Biol..

