# Supplementary material for: Effect of Diphenyleneiodonium Chloride on Intracellular Reactive Oxygen Species Metabolism with Emphasis on NADPH Oxidase and Mitochondria in Two Therapeutically Relevant Human Cell Types
Source: Pharmaceutics. 2020 Dec 23;13(1):10. doi: 10.3390/pharmaceutics13010010 (PMC7823933; doi:10.3390/pharmaceutics13010010)
Supplement: Supplementary file 1 [file pharmaceutics-13-00010-s001.pdf]

pharmaceutics

Supplementary Materials: Effect of Diphenyleneiodonium Chloride on Intracellular Reactive Oxygen Species Metabolism with Emphasis on NADPH Oxidase and Mitochondria in Two Therapeutically Relevant Human Cell Types

Sergejs Zavadskis, Adelheid Weidinger, Dominik Hanetseder, Asmita Banerjee, Cornelia Schneider, Susanne Wolbank, Darja Marolt Presen and Andrey V. Kozlov

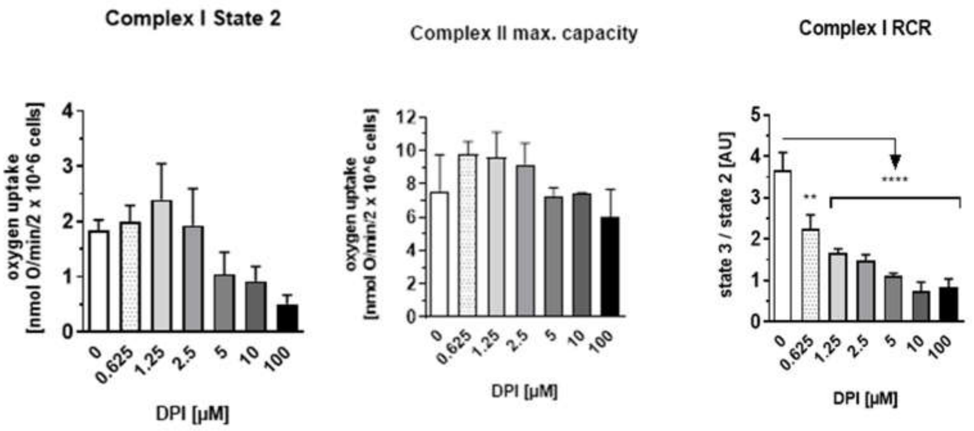

(a) (b) (c)

Figure S1. Effects of diphenyleneiodonium chloride (DPI) on the mitochondrial function in MG-63 cells (**a–c**). (**a**) Effects of diverse DPI concentrations on Complex I-linked State 2 respiration. (**b**) Effects of diverse DPI concentrations on Complex II maximum capacity. (**c**) Effects of diverse DPI concentrations on the Complex I respiratory control ratio (RCR). Data is represented as means + SD (error bars) ($n = 3$). $^{**}p < 0.01$, $^{****}p < 0.0001$. One-way ANOVA followed by multiple comparison post-hoc Fisher's LSD test.

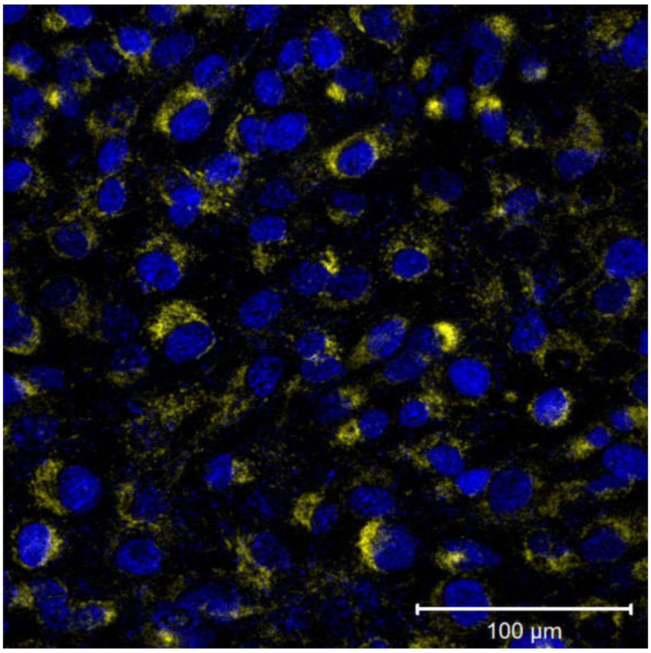

Figure S2. MG-63 cell nuclear and mitochondrial ROS staining in the control cells. Nuclear staining with H2XROS was not detectable. The images were taken with a Zeiss LSM 510 microscope, 10× objective, 5 µM H2XROS (yellow signal) and 3 µM NucSpot® 650 (blue) probe concentrations.

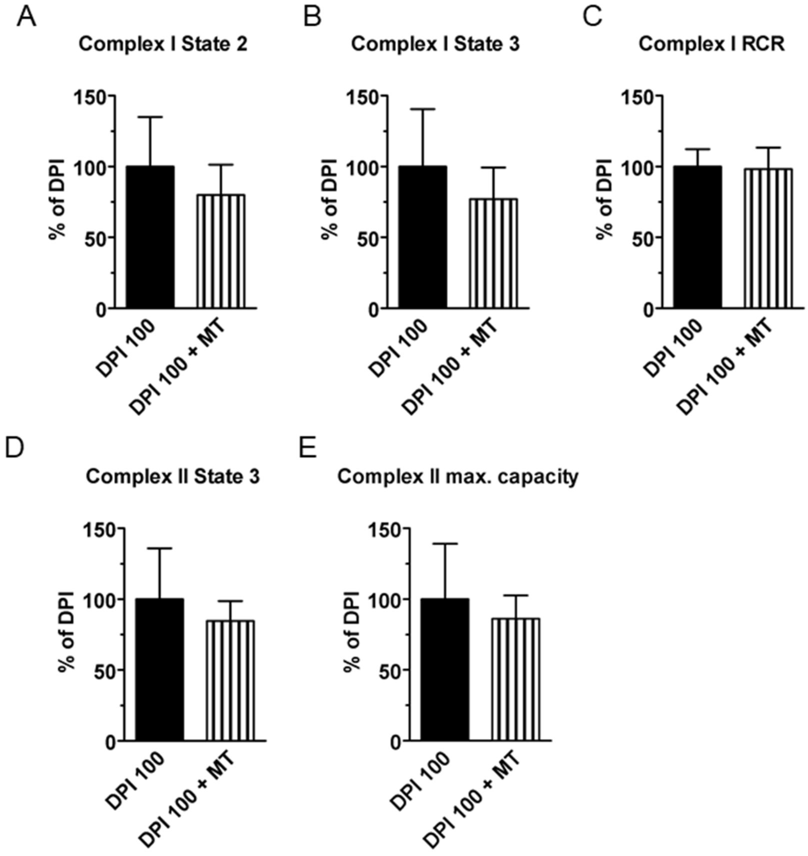

Figure S3. Combined effects of diphenyleneiodonium (DPI) and mitoTEMPO in permeabilized **(A-C)** MG63 cells. (**a**) Combined effects of 100-µM DPI and 20-µM mitoTEMPO on Complex I-linked State 2 respiration (**d**) and Complex II-linked State 3 respiration, (**e**) and the maximum capacity of the Complex II-linked respiration. (**c**) Mean + SD, n = 4.

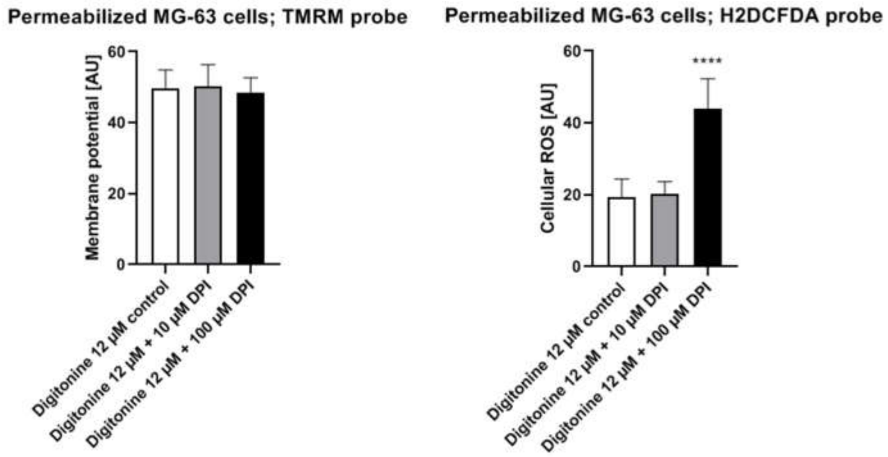

(**a**) (**b**)

Figure S4. Mitochondrial membrane potential and cellular ROS levels in permeabilized MG-63 cells. (**a**) Effects of different concentrations of DPI on the mitochondrial membrane potential in permeabilized MG-63 cells. (**b**) Effects of different concentrations of DPI on cellular ROS in permeabilized MG-63 cells. Represented as means + SD (error bars) (n = 12; each point was obtained by analyzing 2–4 single cells. ****$p < 0.0001$; one-way ANOVA followed by multiple comparison post-hoc Fisher's LSD test.

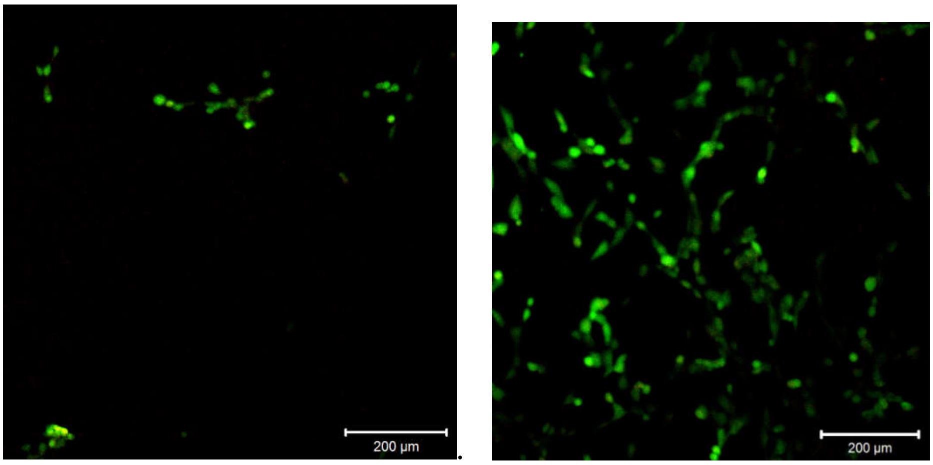

(**a**) (**b**)

Figure S5. Response of permeabilized and nonpermeabilized MG-63 cells to 100 µM DPI. (**a**) Permeabilized cell response to 100 µM DPI. (**b**) Nonpermeabilized cell response to 100 µM DPI. The images were taken with a Zeiss LSM 510 microscope, 10× objective, 50 nM TMRM (red signal) and 10 µM (green signal) H2DCFDA probe concentration.

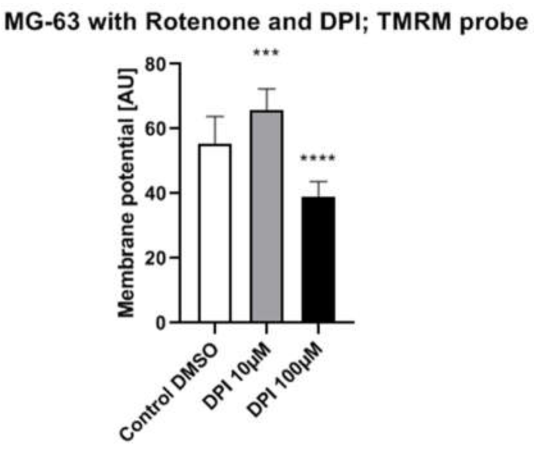

(**a**)

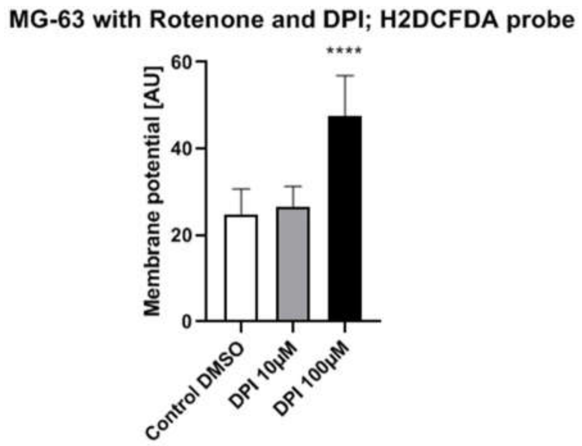

(**b**)

Figure S6. Mitochondrial membrane potential and cellular ROS levels in MG-63 cells exposed to DPI and 5-μM rotenone**.** (**a**) Mitochondrial membrane potential measurement in response to DPI. (**b**) Cellular ROS level measurement in response to DPI. Data is represented as means + SD (error bars) (n = 12; each point was obtained by analyzing 2–4 single cells. ***p < 0.001, and ****p < 0.0001; one-way ANOVA followed by multiple comparison post-hoc Fisher's LSD test.

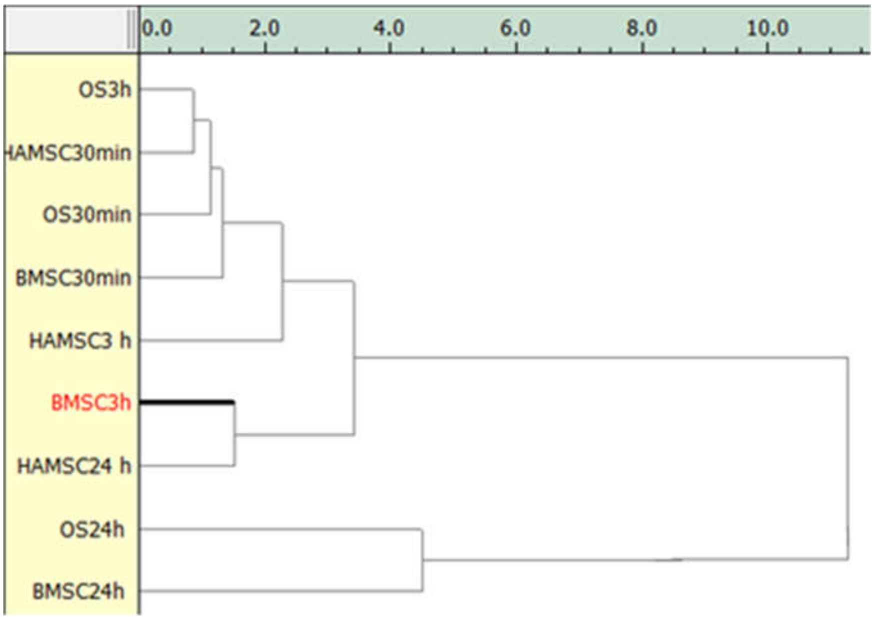

Figure S7. Hierarchical cluster analysis of the hAMSCs, hBMSCs, and osteoblast similarity dendrogram. The clusters defined by this method show similarities between different types of cells at different incubation times based on their averaged fluorescence. The mitochondrial membrane potential and cellular ROS levels were used as predictors for the standardized Ward's cluster analysis. (Epina Datalab 3.5, http://datalab.epina.at/). However, this graph does not indicate the specific characteristics underlying these similarities.
